# Research on Mechanical Properties of Nano-Modified Foam Concrete Improved by Micro-inCorporated Carbon Nanotubes

**DOI:** 10.3390/ma19010184

**Published:** 2026-01-04

**Authors:** Shukun Zhang, Peng Jiang, Haohao Wang, Dianzhi Feng, Hao Wang

**Affiliations:** 1 School of City and Architecture Engineering, Zaozhuang University, Zaozhuang 277160, China; 4254423@163.com (S.Z.);; 2 School of Civil Engineering, Liaoning Technical University, Fuxin 123000, China; 3 College of Pipeline and Civil Engineering, China University of Petroleum (East China), Qingdao 266580, China; 18663670776@163.com

**Keywords:** CNTs, foamed concrete, proportion, mechanical properties of pressure relief filling, support of weak surrounding rock

## Abstract

Foamed concrete is a lightweight, environmentally friendly civil engineering material with excellent absorption capacity. It has been widely applied in engineering fields such as building thermal insulation and pore filling of underground buried pipelines. But the mechanical properties of existing foamed concrete cannot meet the engineering requirements for support, pressure relief and filling of weak surrounding rock. The mechanical properties of foamed concrete were improved with CNTs to prepare CNT foamed concrete (CNTFC) pressure-relieving filling materials. The effects of five factors (the fly ash (FA) incorporation rate, aggregate–cement ratio, water–binder ratio, CNT incorporation rate and foam volume fraction) on the density and 2:1 cylinder strength (the ratio of uniaxial compressive strength to apparent density), splitting tensile (the ratio of splitting tensile strength to apparent density) and specific strength of the CNTFC were analyzed. By combining stress–strain and scanning electron microscopy analyses, the mechanism of improvement of the mechanical strength of CNTFC due to CNTs was clarified. The results show that the foam volume fraction, water–binder ratio and aggregate–cement ratio are the top three factors affecting its strength, followed by the CNT incorporation rate and FA incorporation rate. Among the five influencing factors, only the incorporation of CNTs increases the 2:1 cylinder strength, splitting tensile strength and specific strength. When the doping rate is 0.05%, this ratio specifically refers to the mass of CNTs accounting for 0.05% of the mass of the total cementitious materials of cement and fly ash. At this doping dosage, compared with the condition without CNTs (0% doping dosage), the uniaxial compressive strength increased from 6.23 MPa to 7.18 MPa (with an increase rate of 15.3%). The splitting tensile strength increased from 0.958 MPa to 1.02 MPa (with an increase rate of 6.5%). The density only slightly increased from 0.98 g/cm^3^ to 1.0 g/cm^3^ (with an increase rate of 2.0%), achieving the balance of “high strength-low density”. CNTs and cement hydrates are interwoven into a network structure, and the mechanical properties of the CNTFC are effectively improved by the excellent nanoscopic tensile properties. Excessive doping of CNTs takes 0.05% as the threshold. Exceeding this doping dosage (such as 0.10% and 0.15%) leads to a decrease in its strength and ductility due to CNT agglomeration and deterioration of pore structure. And 0.05% is the ratio of the mass of CNTs to the total cementitious materials of cement and fly ash. At this doping dosage, CNTs are uniformly dispersed and can balance the strength and density of CNTFC. The optimum proportion of CNTs is 0.05%.

## 1. Introduction

There have been some papers studying the characteristics of foamed concrete with low density, good post-peak ductility and strong sealing and absorbing deformation [1,2,3], and it has many applications in building insulation, underground pipeline filling and so on [4,5]. Currently, the industry usually classifies low-density foamed concrete according to apparent density [6]. Ultra-light foamed concrete has high porosity and low thermal conductivity [7] and is mainly used for building thermal insulation layers and lightweight partition walls. Low-density foamed concrete has light weight and certain bearing capacity and is suitable for filling non-load-bearing structures and plugging pipeline pores [8]. Medium-low-density foamed concrete has a better balance between strength and density and can be used for light load-bearing filling or pretreatment of surrounding rock support. The introduction of fly ash and air bubbles greatly reduces the consumption of cement in the process of foamed concrete preparation and also solves the problems of coal ash storage and environmental pollution. By improving the mechanical properties of foamed concrete, the pressure-relief material suitable for supporting soft surrounding rock is improved [9].

At present, researchers have made many achievements in improving the physical and mechanical properties of foamed concrete. For example, previous studies have shown that the incorporation of glazed hollow beads enhances the compressive strength of foamed concrete [10]. Some studies prepared ultra-light foamed concrete with a density of less than 150 kg/m^3^ by the foaming method [11]. Other studies used stone powder as aggregate to replace foamed concrete mixed with river sand to reduce material costs [12]. Additionally, the influence of porosity on the performance of foamed concrete was studied by using the discrete element method [13]. The corrosion resistance of foamed concrete was improved by adding silica fume (SF) [14]. By mixing kenaf fiber [15], the concrete toughness of foamed concrete was improved, and by mixing in carbon nanotubes (CNTs), the flexural strength and compressive strength of autoclaved foamed concrete were increased [16]. Some researchers showed that as a new material [17], foamed concrete has not been widely studied in terms of proportion optimization [18], and the improvement of the performance of foamed concrete is in the exploratory stage. Research on improving the physical and mechanical properties of foamed concrete by nanofiber is even more rare [7]. In this paper, by adding CNTs, the nanoscale and tensile strength properties of foamed concrete are improved, and the mechanical properties of foamed concrete are effectively improved [19,20]. A CNT foamed concrete (CNTFC) filling medium suitable for unloading surrounding rock is prepared. The mechanical and microscopic characteristics of CNTFC with different proportions are analyzed by the orthogonal test method. The optimal proportioning of CNTFC is determined by comprehensive analysis according to the actual control requirements of surrounding rock deformation [21,22,23,24].

## 2. Experimental Design and Specimen Preparation of CNT Foamed Concrete (CNTFC) as Pressure-Relief Filling Material

### 2.1. Constituents

**Figure 1:**
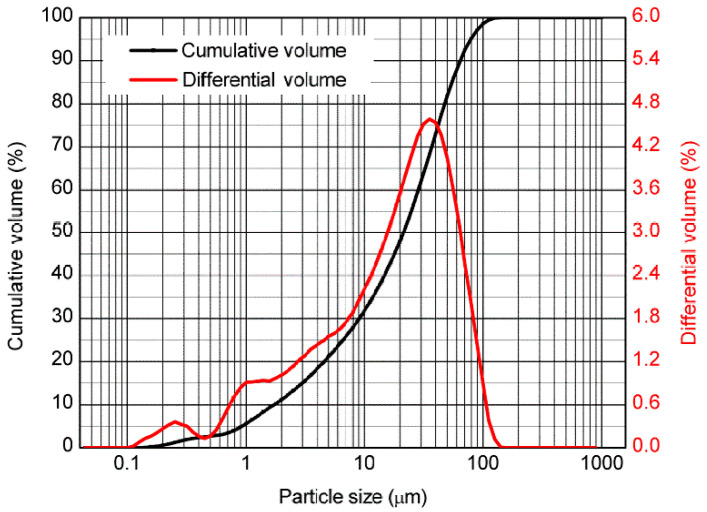
Particle size curve of FA [26].

The cement was Fuxin Yingshan ordinary Portland cement PO42.5 according to national standards GB/T12960 [25] the supplemental fly ash (FA) was produced by Fuxin Power Plant, with a density of 2.12 g/cm^3^, a fineness of 10.8% and a quality grade of I. This curve was plotted using the laser particle size analysis method. “Differential volume” refers to the volume during particle size analysis. The percentage of the volume of fly ash particles within a specific particle size range (such as 0.1~1 μm, 1~10 μm, 10~100 μm, etc.) accounts for the total volume of all fly ash particles. It reflects the particle size characteristics of fly ash: “dominated by fine particles, concentrated gradation, and few coarse particles”. The grading curve is shown in Figure 1. The aggregate was a natural river sand with a particle size less than 2.36 mm after screening, and its apparent density was 2.23 g/cm^3^. The liquid was produced by Zhengzhou Pengyi Chemical Products Co., Ltd. (Zhengzhou, China). ZT-F50 type cement has a foaming volume of 60 mL/g, an active substance content of 30% and a decomposition temperature of 5 °C. The surface tension at 25 °C is 28–32 mN/m, and it can effectively reduce the surface energy of aqueous solution and promote the formation of uniform bubbles. The surfactant composition is dominated by anionic surfactant (sodium dodecylbenzene sulfonate, mass fraction 15–20%) and blended with nonionic surfactant (fatty alcohol polyoxyethylene ether, mass fraction 5–8%). The synergistic effect of the two can improve foam stability. The CNTs were produced by Shenzhen Turing Evolution Technology Co., Ltd. (Shenzhen, China). The CNTs are multi-walled carbon nanotubes (MWCNTs), treated by mixed acid ultrasonic oxidation. Their surface is grafted with carboxyl (–COOH) and hydroxyl (–OH) groups. The total content of functional groups is 2.5–3.0%, and it can improve interface compatibility with cement paste and reduce agglomeration. The residual catalyst is Fe and Ni metal particles, with a mass fraction ≤0.5%. The parameters of the CNTs are shown in Table 1. The chemical compositions of materials are shown in Table 2.

### 2.2. Test Specimen Preparation

The effect of CNT dispersion on the mechanical properties of foamed concrete is very important, and CNT dispersion mainly comprises physical dispersion and chemical dispersion [27]. The study used ultrasonic dispersion plus physical stirring to disperse CNTs. First, the CNTs were wetted in a closed plastic container, and then the CNTs were mechanically stirred and ultrasonically dispersed for 30 min to prepare a CNT dispersion. The slurry mixture was prepared by thoroughly mixing cement, FA, natural sand and water according to the selected ratio [26]. The foaming agent and water were placed in a deep plastic bucket at a ratio of 1:40 and mechanically stirred with a stirrer rotating at 1500 r/min to prepare a uniform and stable foam. The prepared CNT dispersion was mixed into the slurry mixture, stirred for 10 min, mixed with foam for approximately 4 min, and allowed to stand for 2 to 3 min. Then, the foamed concrete sample was poured. The cast specimens were cylindrical, with a diameter of 5 cm and a height of 10 cm. After standing for 48 h in 20 °C with the test model covered with wet cloth, each specimen was demolded, placed in a curing box for standard curing for 28 days, removed to air dry for 3 h, and then subjected to mechanical tests. The strength test was performed according to the relevant specifications [28].

### 2.3. Test Scheme

**Figure 2:**
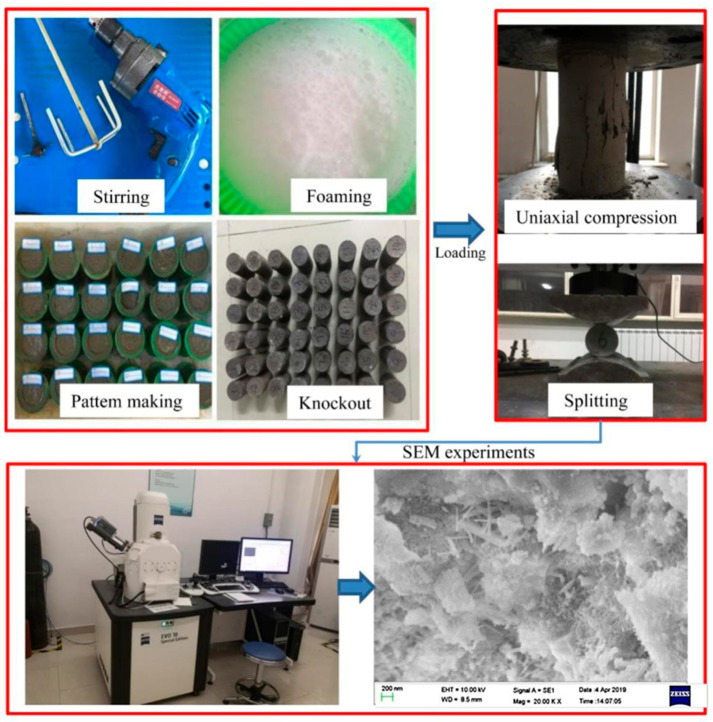
Specimen preparation and testing process.

Based on the material performance requirements of roadway support and pressure relief, an orthogonal test was designed to measure the CNTFC specimens with different FA incorporation rates, aggregate–cement ratios, water–binder ratios, CNT incorporation rates and foam volume fractions. The uniaxial compression strength, splitting tensile strength and density data are used to analyze the influence of various factors on the performance of CNTFC and initially determine the proportioning scheme applicable to the surrounding rock support and roadway pressure relief. The device for the characterization test is an in situ transmission electron microscope. The device used for the characterization test is a JSM-7500F scanning electron microscope (SEM), which is available at Liaoning Technical University (Fuxin, Liaoning Province, China). The manufacturer of this SEM is JEOL Ltd., a well-known equipment supplier in the field of electron microscopy, and its headquarters is situated in Tokyo, Japan. The image quality is clear. To save costs, no metal plating was conducted before the SEM analysis. To analyze the internal structure and pore characteristics of the corresponding CNTFC specimens, we used SEM technology with medium voltage (5–10 kV) and medium WD (8–12 mm), which laid the foundation for the subsequent determination of the optimal mixing ratio. The specific test specimen preparation and testing process are shown in Figure 2.

Some studies indicate that it is unable to provide a surrounding rock support strength close to 1 MPa [29,30]. Considering the stress concentration that arises in the high-strength support structure, researchers showed that the required strength for the performance of the surrounding rock support pressure-relief material is between 1~10 MPa [31,32]. Compared with traditional concrete, foamed concrete has the characteristics of large deformation, light weight and low cost, and the excellent plasticity of foamed concrete can be used as a large deformation absorption material [33,34,35]. CNTs are high-strength and high-toughness materials with tensile strength of 50–200 GPa and elastic modulus of about 1 TPa. In recent years, some scholars have proposed to improve foamed concrete by using CNTs [17]. According to the literature and the preliminary research results of this research group, natural sand is used as the aggregate, FA and cement are used as the cementing materials and CNT fibers are micro-doped to prepare CNTFC filling and pressure-relief material. A 5-factor, 4-level orthogonal test was designed. The specific ratio scheme is shown in Table 3.

The FA incorporation rate is the mass ratio of the FA to that of the cement. The aggregate–cement ratio is the mass ratio of the natural sand to that of the cementitious material. The water–binder ratio is the mass ratio of the water to that of the cementitious material. The CNT incorporation rate is the mass of the CNTs to that of the glue. The amount of foam is the ratio of the volume of the foam to the volume of the mixture.

The tests were carried out at the Structural Laboratory of the School of Civil Engineering, Liaoning Technical University. The loading test instrument employed in this study is a WDW-300KN electronic universal testing machine, which is produced by Jinan Times Tester Co., Ltd. (Jinan, Shandong Province, China). The test was carried out using this equipment housed at Liaoning Technical University (Fuxin, Liaoning Province, China). The loading rate was controlled to 0.05 MPa/s during the splitting tensile strength tests, and the loading rate during the 2:1 cylinder strength tests was controlled to 1 mm/min. The device for characterization tests is an in situ transmission electron microscope. A commissioned third party, namely “Testing Dog” (a professional testing service platform in China), performed the microstructure testing. The SEM device used for the test was model SUPRA55, which is manufactured by Carl Zeiss AG (Oberkochen, Germany). This equipment has a resolution of 1.0 nm and a magnification range of 12~900,000×.

## 3. Analysis on Influence Rules and Mechanisms of CNTs on Mechanical Properties of CNTFC

### 3.1. Strength and Density Test Results

The 2:1 cylinder strength, splitting tensile strength and density test results of the CNTFC filling and pressure-relieving material after curing for 28 days are shown in Table 4. Each result is accurate to the third decimal place.

### 3.2. Visual Analysis

The specimens were divided into two large groups, and 16 proportioned specimens were prepared for each large group; three specimens with the same ratio were processed for parallel testing. A total of 96 specimens was prepared. The 2:1 cylinder strength and splitting tensile strength tests with five factors and four levels were carried out to obtain the corresponding stress–strain data; additionally, the specimen density was measured.

Table 4 shows that the #6 specimen has the highest 2:1 cylinder strength, and the combination ratio is A_2_B_2_C_1_D_4_E_3_. The corresponding FA incorporation rate is 60%, aggregate–cement ratio is 5%, water–binder ratio is 45%, CNT incorporation rate is 0.15% and foam volume fraction is 120%; the corresponding splitting tensile strength ranks fourth in the 16-group test, and the corresponding density ranks third in the 16-group trial. The #8 specimen has the highest splitting tensile strength and density, and the combination ratio is A_2_B_4_C_3_D_2_E_1_, corresponding to an FA incorporation rate of 60%, an aggregate–cement ratio of 15%, a water–binder ratio of 55% and a CNT incorporation rate of 0.05%. The corresponding foam volume score is 50%. The 2:1 cylinder strength of the #8 specimen ranks fourth in the 16 groups of tests. Specimens #5, #13 and #14 have the lowest densities, and specimen #5 is directly eliminated due to not only its low strength index but also its low FA incorporation rate, high CNT incorporation rate and poor economy and applicability. Although the FA incorporation rates of specimens #13 and #14 are high and their CNT ratios are low, their mechanical strength indexes are low, and it is difficult to meet the pressure-relief requirements of the surrounding rock support. These combinations are also directly excluded.

### 3.3. Orthogonal Test Range Analysis

The extreme difference between the 2:1 cylinder strength and splitting tensile strength shown in Table 5 suggests that the foam volume fraction, the water–binder ratio and the aggregate–cement ratio affect the strength of the test specimens the most, while the CNT incorporation rate and FA incorporation rates have a secondary effect. This result is because the foam volume fraction and the water–binder ratio determine the porosity of a test specimen. The lower the porosity of a test specimen, the higher the density and the more favorable the corresponding strength parameter, significantly affecting the 2:1 cylinder strength and splitting tensile strength. And the higher strength parameters of specimens correspond with higher 2:1 cylinder strength and splitting tensile strength. Table 4 also proves this point. Porosity is a core intermediate factor linking CNTFC’s mix parameters to its final properties, exerting three key effects. For strength, it correlates negatively—when the foam volume fraction (key for porosity control) rises from 50% to 150%, 2:1 cylinder strength drops by 54.2% and splitting tensile strength by 60.4%, and excessive porosity disrupts CNT-hydrate networks, further weakening strength. For density, it also correlates negatively (air in pores is far less dense than a solid matrix like cement/fly ash)—the foam volume fraction 50%→150% cuts density by 26.74%, while the water–binder ratio 45%→60% reduces it by 27.12%. For ductility/toughness, moderate porosity boosts ductility via deformation space, but excess causes brittle failure; only when porosity matches 0.05% CNT incorporation (CNTs fill small pores and inhibit cracks) can CNTFC balance strength, density and ductility. The subsequent specific strength analysis shows this in detail. At present, it is difficult to further improve the mechanical properties and economy of foamed concrete by adjusting factors such as the foam volume fraction, water–binder ratio and aggregate–cement ratio. The incorporation of FA can adjust the gradation of the mixture and replace part of the cement to achieve economic efficiency. However, due to its low activity, increasing the dosage will reduce the strength of the CNTFC. Therefore, the incorporation of CNTs is required to improve the micro-scale characteristics of foamed concrete, which further improves the mechanical properties of foamed concrete.

### 3.4. Influence Analysis of the Strength and Density of CNT Foamed Concrete

#### 3.4.1. Analysis of Compressive Strength

**Figure 3:**
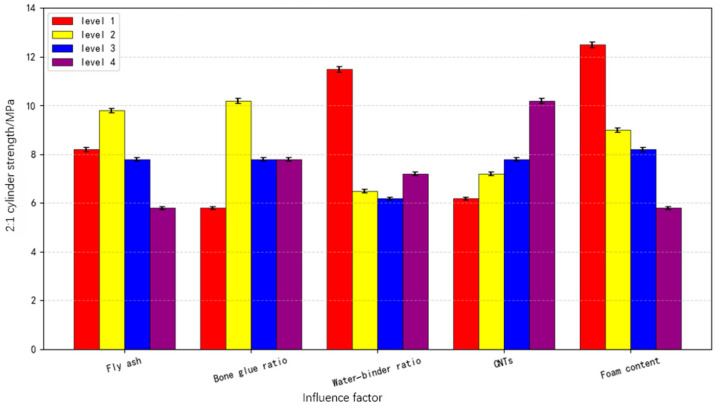
Variation in CNTFC 2:1 cylinder strength under the influence of various factors.

According to the orthogonal test method, we calculated the average value of the 2:1 cylinder strength of CNT foamed concrete (CNTFC) at 28 days under each level of each influencing factor from the visual analysis table (Table 4) and also drew a column chart (Figure 3). When the fly ash (FA) incorporation rate increases from 40% to 60%, the 2:1 cylinder strength of CNTFC rises from 8.180 MPa to 9.675 MPa, with an increase rate of 18.28%; when it increases from 60% to 80%, the strength drops to 7.832 MPa, with a decrease rate of 18.5%; when it increases from 80% to 100%, the strength drops to 5.686 MPa, with a decrease rate of 27.40%. The reason for these changes is the “morphological effect” of the glass beads in the lesser amount of FA. It promotes the deflocculation effect during the initial cement hydration. Additionally, the microbeads and small particles can gather in dense clusters outside the foam pores. The other pores, together with the cement gel, form a dense bubble wall that increases the strength of the CNTFC. The FA incorporation rate is further improved. Because its activity is lower than the cement activity, using FA in place of an equal amount of cement reduces the amount of cement required, delays the hydration degree of the whole system, and leads to a decrease in the 2:1 cylinder strength of the CNTFC. When the aggregate–cement ratio ranges from 0 to 15%, the 2:1 cylinder strength of the CNTFC first increases and then decreases. The corresponding strengths are 5.607 MPa, 10.117 MPa, 7.855 MPa and 7.795 MPa. When the aggregate–cement ratio is 5%, the 2:1 cylinder strength of the CNTFC is the highest. When the incorporation rate of natural sand (used as aggregate) is low, the sand is surrounded by FA, cement and air bubbles. At this time, the strength of the CNTFC mainly depends on the strength of the FA and cement slurry, so the strength is low. As the aggregate–cement ratio increases, cement and FA encapsulate the sand aggregate. They form a bearing matrix around the bubbles. Thus, the CNTFC achieves higher compressive strength. When the sand ratio is high, the hydrated products from FA and cement are less likely to surround the aggregate particles, and inclusions are difficult to carry within the air bubbles, resulting in a decrease in the strength of the CNTFC.

When the water–binder ratio is 45%, the 2:1 cylinder strength of the CNTFC is the highest, being 11.541 MPa. When the water–binder ratio is increased from 45% to 50%, the 2:1 cylinder strength of the CNTFC drops sharply by 43.47%. When the water–binder ratio is between 50% and 60%, the strength regularity of the CNTFC is not obvious. Low-water cement has a higher strength than CNTFC, high-water cement has a lower strength than CNTFC and the effect is consistent with that of ordinary concrete. When the water–binder ratio exceeds 50%, the influence on CNTFC is not obvious, and the strength is at a low level, which may be related to the CNTFC solidification and curing.

When the CNTs increased from 0 to 0.15%, the 2:1 cylinder strength of the CNTFCs showed a stepwise increase, and the corresponding strengths were 6.232 MPa, 7.185 MPa, 7.652 MPa and 10.304 MPa, with growth rates of 15.29%, 6.50% and 34.66%, respectively. CNTs, by virtue of their excellent tensile properties, are intertwined with the cement, FA and hydrates and enclose the aggregate more densely, improving the foamed concrete characteristics and thus effectively improving the strength of the foamed concrete. Foam incorporation is the most significant factor affecting the strength of CNTFC. As the foam incorporation increases from 50% to 150%, the 2:1 cylinder strength of CNTFC decreases: 12.666 MPa, 9.794 MPa, 8.117 MPa and 5.796 MPa, corresponding to decreases of 22.67%, 17.12% and 28.59%, respectively. The introduction of bubbles increases the void space, reduces the load-carrying capacity of the cement and aggregate and greatly reduces the strength of the CNTFC.

**Figure 4:**
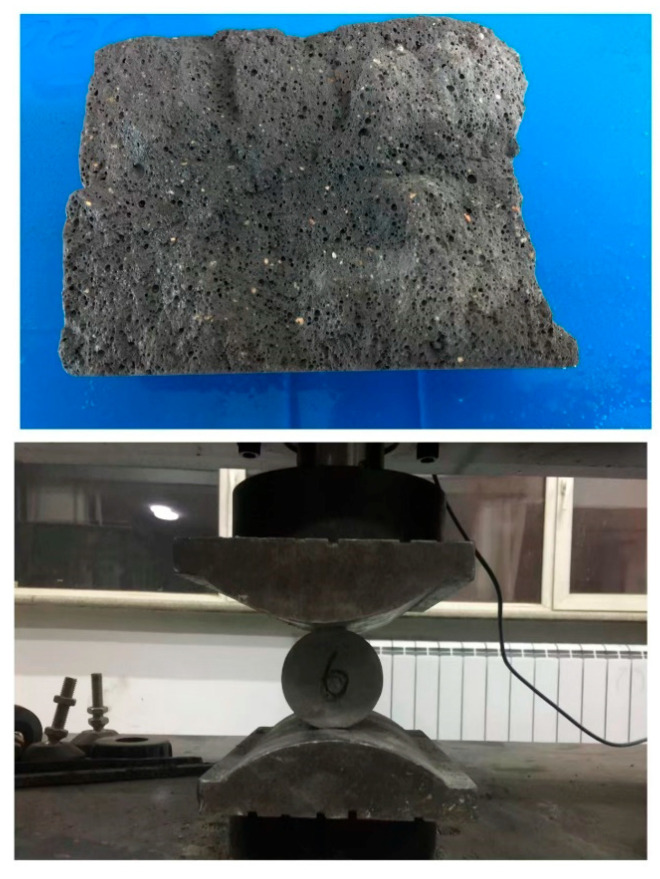
Splitting test diagram.

Based on the density test results in the paper (Table 4, Figure 4) and combined with the classic correlation formula between porosity and density of foamed concrete [13,17],

(1) $$P=1-\frac{\rho }{\rho_{0}}$$

where P is the total porosity (%), ρ (g/cm^3^) is the measured density of CNTFC (Table 4), and ρ_0_ (g/cm^3^) is the solid-phase density of the matrix (based on the chemical composition of cement and fly ash in Table 2). The calculated value of ρ_0_ is 2.28 g/cm^3^. Thus, the actual porosity is estimated. Taking the groups with a foam volume fraction of 50%, a foam volume fraction of 150%, a water–binder ratio of 45% and a water–binder ratio of 60% separately, we calculate their average densities to be 1.253 g/cm^3^, 0.918 g/cm^3^, 1.180 g/cm^3^ and 0.860 g/cm^3^, respectively. According to the formula, the porosity of the group with a foam volume fraction of 50% is 45.04%. The porosity of the group with a foam volume fraction of 150% is 60.09%; the porosity of the group with a water–binder ratio of 45% is 48.25%. The porosity of the group with a water–binder ratio of 60% is 62.28%. The results are highly consistent with the conclusion that “foam volume fraction and water–binder ratio are the primary factors affecting strength”. This verifies the causal relationship that “porosity regulates strength as an intermediary variable”.

#### 3.4.2. Splitting Tensile Strength Analysis

**Figure 5:**
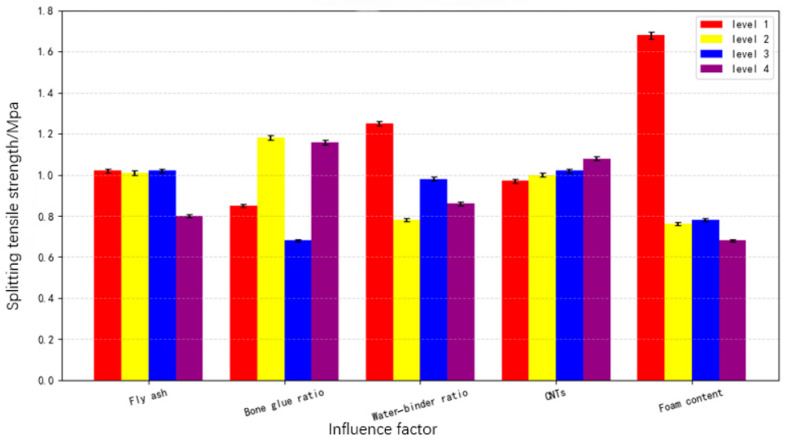
Variation in CNTFC splitting tensile strength under the influence of various factors.

**Figure 6:**
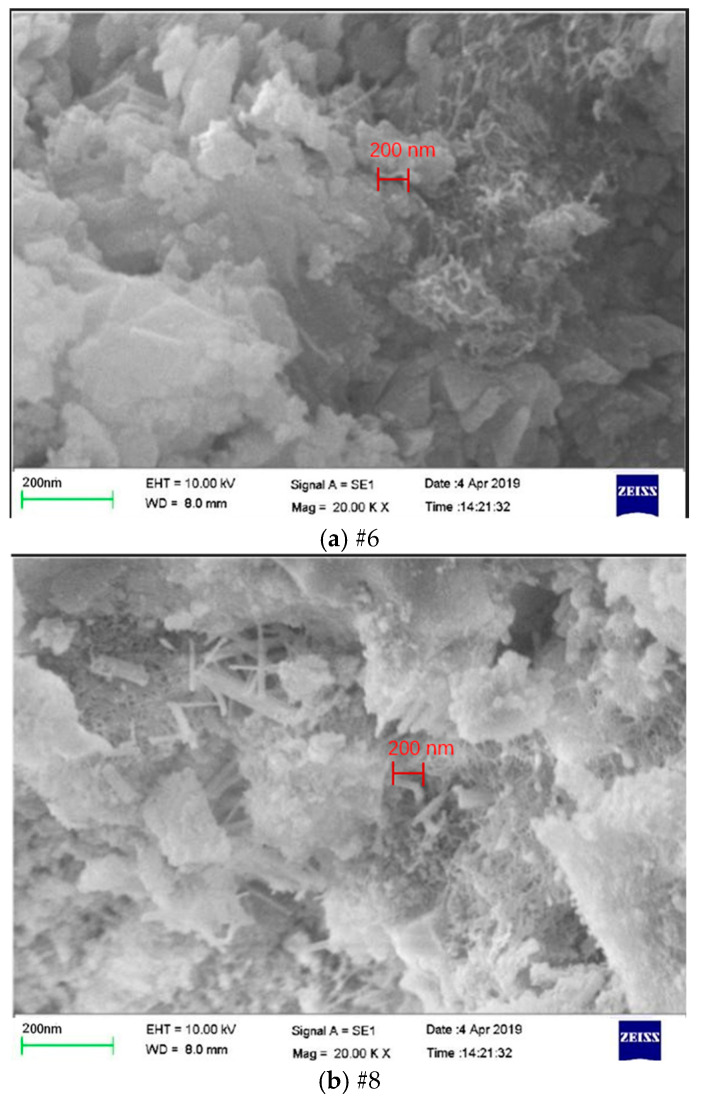
SEM of CNTFC specimens (200 nm).

**Figure 7:**
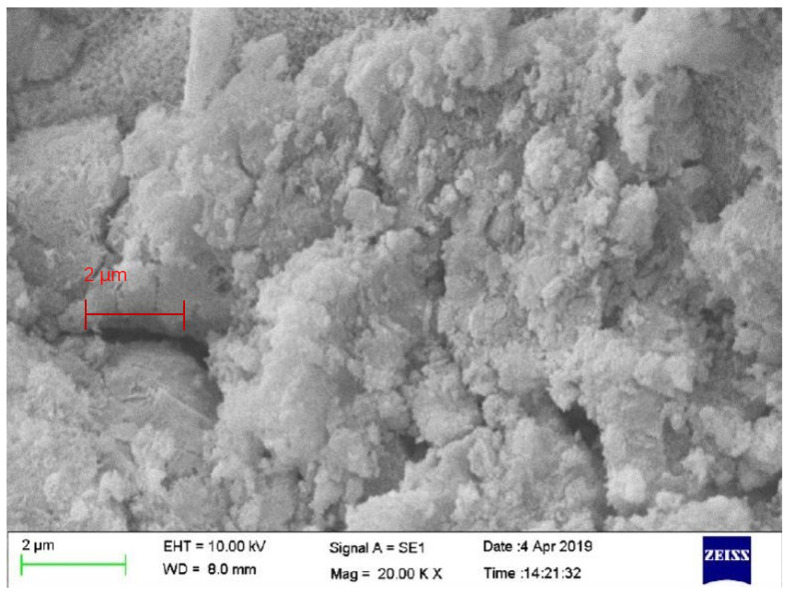
SEM of CNTFC specimen (2 μm #13).

According to the orthogonal test method, the average value of the 28-day splitting tensile strength index at each level of each factor is obtained from the visual analysis table, and a columnar section was drawn, as shown in Figure 5. When the FA incorporation rate is 40%, 60% and 80%, the CNTFC splitting tensile strength is 1.023 MPa, 1.013 MPa and 1.014 MPa, respectively, and the relationship between these two parameters is not obvious. When the FA incorporation rate is increased from 80% to 100%, the CNTFC splitting tensile strength is reduced to 0.786 MPa. When the FA incorporation rate is less than 80%, it has no obvious effect on the tensile properties of the cement. When the FA incorporation rate reaches 100%, the splitting tensile strength of the CNTFC is similar to the 2:1 cylinder strength, a decrease of 22.49% from the latter result. This trend is similar to that of the 2:1 cylinder strength. The activity of FA is lower than the activity of cement. The replacement of cement by the same amount of FA reduces the amount of cement, delays the hydration degree of the whole system, and thus causes the CNTFC splitting tensile strength to decrease. When the aggregate–cement ratio ranges from 0 to 15%, it is 0.836 MPa, 1.174 MPa, 0.669 MPa and 1.157 MPa, respectively. The trend of the variation in the CNTFC splitting tensile strength shows disordered fluctuation, indicating that the sand aggregate contributes only slightly to the tensile strength of the matrix. The formula for specific fracture energy (Gf) [34] is

(2) $$G_{f}=\frac{w}{A}=\frac{\int_{0}^{\varepsilon }\sigma \left(\varepsilon \right)\mathrm{d}\varepsilon }{A}V$$

where $\int_{0}^{\varepsilon }\sigma \left(\varepsilon \right)\mathrm{d}\varepsilon$ is the area under the stress–strain curve (J/m^3^), V is the sample volume (m^3^) and A is the fracture area (m^2^). According to the formula, the specific fracture energy of Sample #8 (with a CNT content of 0.05% and a sand–binder ratio of 15%) is calculated to be 128 J/m^2^, and the splitting tensile strength is 1.997 MPa. Sample #12 (without CNTs, with a sand–binder ratio of 15%) has a specific fracture energy of 86 J/m^2^ and a splitting tensile strength of 0.804 MPa. Sample #11 (with a CNT content of 0.05% and a sand–binder ratio of 10%) has a specific fracture energy of 115 J/m^2^ and a splitting tensile strength of 0.786 MPa. Results show that when the sand–binder ratio increases from 5% to 15%, the specific fracture energy only increases by 8.2%. It verifies the conclusion that “sand has little contribution to splitting tensile strength”. Combined with SEM images (Figure 4, Figure 6 and Figure 7), we deduce the macroscopic fracture mode: Groups without CNTs/low CNT content: fracture occurs along the aggregate–matrix interface, which is brittle fracture, with a flat fracture surface. Group with a CNT content of 0.05%: CNTs interweave with hydration products to form a network structure; fracture needs to overcome the bonding force of CNT bridging, which shows ductile fracture, with a serrated fracture surface. High CNT content group (such as Sample #6, 0.15%): CNT agglomeration causes local stress concentration; fracture propagates along the edges of agglomerates, and toughness decreases. This is consistent with the result that “the growth rate of splitting tensile strength decreases”.

When the water–binder ratio is 45%, the CNTFC splitting tensile strength is the highest, at 1.260 MPa. When the water–binder ratio is increased from 45% to 50%, the splitting tensile strength of the CNTFC increased by 40.43%. When the water–binder ratio is between 45% and 60%, the regularity of the CNTFC splitting tensile strength is not obvious. The effect of the water–binder ratio on the splitting tensile strength is similar to that on the 2:1 cylinder strength, indicating that a 45% water–binder ratio has the best effect on FA and cement hydration. When the amount of CNTs is increased from 0 to 0.15%, the splitting tensile strength of the CNTFC generally increases, and the corresponding strengths are 0.964 MPa, 1.016 MPa, 1.002 MPa and 1.053 MPa, respectively. The growth rate is not high, showing that the increase in the macroscopic tensile properties of CNTs in foamed concrete is not significant.

The foam incorporation is the most significant factor affecting the CNTFC splitting tensile strength. As the foam incorporation increases from 50% to 80%, the CNTFC splitting tensile strength decreases by 55.04%, from 1.675 MPa to 0.753 MPa. As the foam incorporation increases from 80% to 150%, the CNTFC splitting tensile strength decreases, from 0.753 MPa to 0.745 MPa to 0.664 MPa, and the splitting tensile strength decreases by 1.06% and 10.87%, respectively. The introduction of bubbles increases the void space in the CNTFC and reduces the bearing capacity of the cement and aggregate. In particular, the splitting tensile strength of CNTFC is greatly reduced when the foam incorporation is increased from 50% to 80%.

#### 3.4.3. Density Analysis

**Figure 8:**
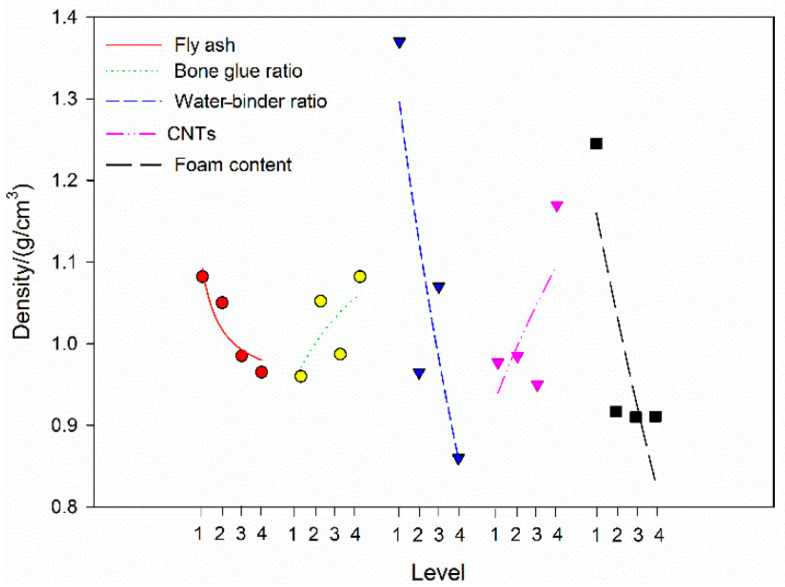
CNTFC density changes under the influence of various factors.

According to the orthogonal test method, the average value of the 28-day density index at each level of each factor is obtained from the visual analysis table. To facilitate trend analysis, a function is used to fit the trend of the discrete points, as shown in Figure 8. When the FA incorporation rate increases from 40% to 100%, the apparent density of the CNTFC decreases by 10.90% from 1.083 g/cm^3^ to 0.965 g/cm^3^. The overall decline is relatively small. The density of FA is slightly lower than the density of cement. As the replacement rate increases, the overall strength of the specimen decreases, but the magnitude of the decrease is not considerable. As the water–binder ratio and foam production increase, the density also decreases at a higher rate of decrease. When the water–binder ratio increases from 45% to 60%, the density decreases by 27.12% from 1.180 g/cm^3^ to 0.860 g/cm^3^. When the foam volume fraction increases from 50% to 150%, the density decreases by 26.74% from 1.253 g/cm^3^ to 0.918 g/cm^3^. The water and gas densities are much lower than the density of the mixture, and the increases in the water–binder ratio and the amount of foam greatly reduce the density of the specimen. When the aggregate–cement ratio increases from 0 to 15%, the density increases by 13.54% from 0.960 g/cm^3^ to 1.090 g/cm^3^. When the incorporation of CNTs is increased from 0 to 0.15%, the density increases by 18.92% from 0.978 g/cm^3^ to 1.163 g/cm^3^. The incorporation of aggregate cement, compared to that of CNTs, increased the density of the specimen, but the increase was relatively small because when preparing CNTFCs, it is necessary to add natural sand (as an aggregate) and CNTs to improve the CNTFC characteristics and prepare lightweight concrete with a certain strength. Therefore, the amount of sand and CNTs with a higher density is controlled, and the density of the CNTFC is not substantially increased.

Symbols and lines in the figure correspond to different influencing factors of CNTFC density: solid line for fly ash incorporation rate, circle + dot line for aggregate–cement ratio, short dash line for water–binder ratio, triangle + dash-dot line for CNT incorporation rate and long dash line for foam volume fraction.

#### 3.4.4. Specific Strength Analysis

**Figure 9:**
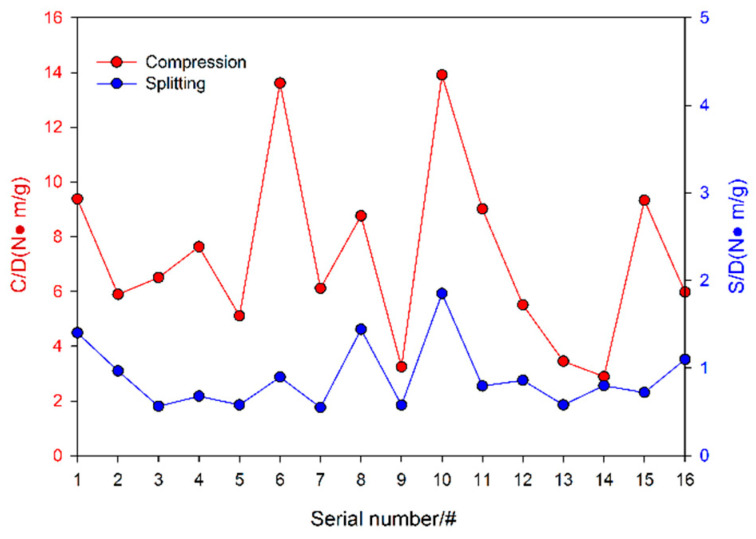
Different ratios of CNTFC specific strength.

Low density and high strength are ideal roadway surrounding rock support pressure-relief material characteristics. The specific strength is an important index used to measure the performance of filling materials [35], and there are few studies on the specific strength of foamed concrete materials. The specific strength curve is plotted by calculating the ratio of 2:1 cylinder strength, tensile splitting strength and apparent density for each ratio, as shown in Figure 9. C/D is the compressive specific strength (the ratio of uniaxial compressive strength to apparent density). S/D is the splitting tensile specific strength (the ratio of splitting tensile strength to apparent density). For CNTFC, the specific strength is approximately the same as the splitting tensile strength because these strengths are both a function of a basic fixed coefficient [18], and the trends of the curves formed by considering the same density ratio are similar. The average compressive strength of the prepared CNTFC is 8.472 N·m/g, and the average splitting tensile strength is 0.903 N·m/g. The cylinder strength of the #6 specimen reaches 15.194 N·m/g, which is 27.32% higher than the optimum specific strength of 11.960 N·m/g recorded [35], and the corresponding ratio combination is A_2_B_2_C_1_D_4_E_3_; the specific strength of the 2:1 cylinder strength of the #4 specimen is 3.616 N·m/g, and the corresponding mix proportion is A_1_B_4_C_4_D_4_E_4_; the tensile strength of the #10 specimen reaches 1.850 N·m/g, and the tensile strength of the #7 specimen is 0.547 N·m/g. The corresponding mix proportions are A_3_B_2_C_4_D_3_E_1_ and A_2_B_3_C_4_D_1_E_2_.

**Figure 10:**
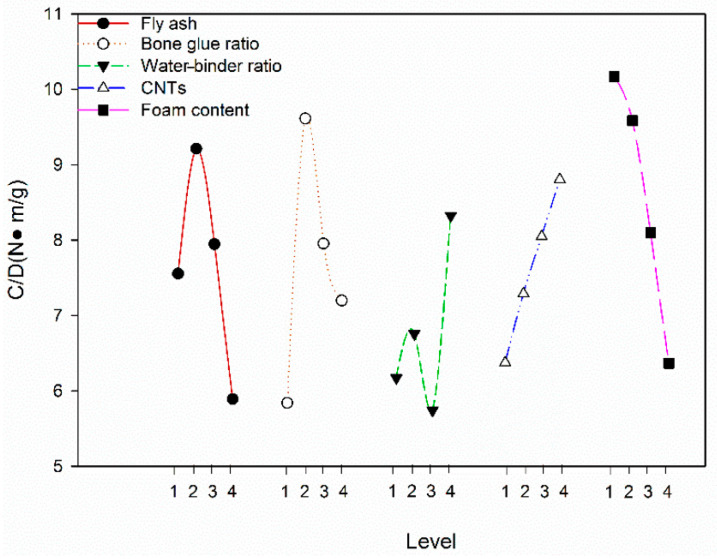
The average value of the strength-to-density ratio index of a cylinder under different factors 3.5. Influence of CNT Content on CNTFC Performance.

Since the 2:1 cylinder strength has an intrinsic relationship with the splitting tensile strength, only the CNTFC compressive strength at each level of each influencing factor is analyzed. According to the orthogonal test method, the average value of the 28-day 2:1 cylinder strength and density ratio index at each level of each factor is obtained from the visual analysis table, and the function is used to fit the trend of the discrete points for the trend analysis, as shown in Figure 10. When the FA incorporation rate increases from 40% to 100%, the CNTFC compressive specific strength curve trend first increases and then decreases, and 60% is the optimal incorporation rate. As the FA incorporation rate is increased from 40% to 60%, the CNTFC strength increases, and the FA is used to replace the cement to reduce the density of the mixture; when the FA incorporation rate increases from 60% to 80%, the strength and density of CNTFC decrease, and the strength decreases more. When the aggregate–cement ratio increases from 0 to 15%, the strength trend first increases and then decreases. When the aggregate–cement ratio is 5%, the 2:1 cylinder strength of the CNTFC is the highest; excessive added aggregate causes a decrease in the strength of the CNTFC and a considerable increase in density, so the specific strength curve decreases.

When the water–binder ratio is increased from 45% to 55%, the strength and density of CNTFC decrease, but the decrease is not large, so there is no obvious regularity in the specific strength trend; when the water–binder ratio is increased from 55% to 60%, the CNTFC density is more reduced with respect to the strength, resulting in a sharp increase in the specific strength curve. When the CNT doping amount is increased from 0 to 0.15%, the CNTFC specific strength curve tends to increase sharply because the micro-doped CNTs have little effect on the CNTFC density but can greatly increase the 2:1 cylinder strength.

The specific strength curve under the influence of the increase in foam incorporation exhibits a steep drop. For the specific strength, the introduction of bubbles causes the 2:1 cylinder strength of the CNTFC to further decrease. To obtain low-density, high-strength CNTFC materials, in addition to controlling the conventional FA incorporation rate, aggregate–cement ratio and water–binder ratio, it is necessary to incorporate materials that can have a small effect on density but greatly increase the strength of concrete. CNTs can satisfy this requirement.

### 3.5. Influence of CNT Content on CNTFC Performance

The above analysis shows that among the five factors of the FA incorporation rate, aggregate-cement ratio, water-binder ratio, CNT incorporation rate and foamed volume fraction, the incorporation of FA and foamed can reduce the cost of concrete but also reduce its overall mechanical properties. The increase in water-binder ratio decreases the overall strength, and a suitable aggregate-cement ratio can effectively increase the CNTFC specific strength. Only the incorporation of CNTs increases the 2:1 cylinder strength, splitting tensile strength and specific strength of the CNTFC, and a suitable incorporation amount allows the CNTFC to maintain a lower density level while maintaining a higher strength. Based on this, a hypothesis is proposed that in the CNT foamed concrete (CNTFC) surrounding rock pressure relief filling material, micro-dosage carbon nanotubes (CNTs) (dosage range of 0–0.15%) can intertwine with cement and fly ash hydration products to form nanoscale bridging and spatial network structures, it can effectively inhibit the initiation and propagation of cracks. Within this dosage range, as the CNTs dosage increases, the material density increases moderately (with an increase rate ≤16.2%), and its uniaxial compressive strength, splitting tensile strength and specific strength increase simultaneously. When the CNTs dosage is 0.05%, the above mechanical indicators are comprehensively optimal. If the CNTs dosage exceeds 0.05%, CNTs tend to agglomerate, which will expand the medium transition zone and weaken the bridging and nucleation effects, ultimately leading to a decrease in the material’s strength and ductility instead. It is concluded that CNTs improve the microscopic characteristics and nanoscale tensile properties of the mixture and effectively inhibit the initiation and expansion of fractures. Therefore, to study the mechanism of the overall improvement in the mechanical performance of CNTFC, the CNTFC ultimate strain improvement and micro-scale properties are further studied [36].

#### 3.5.1. Analysis of the Effect of the Ultimate Strain on Improvement

**Figure 11:**
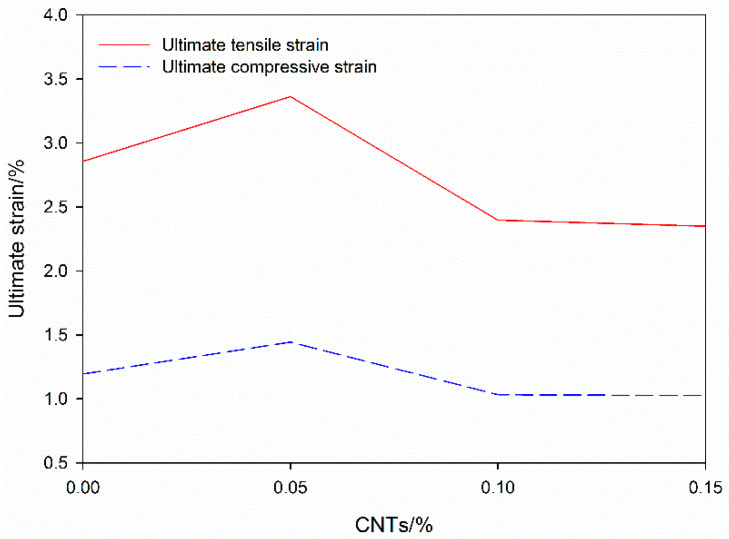
CNTFC ultimate strain under the influence of different CNT incorporation rates.

According to the ultimate strain value corresponding to the stress–strain curve of each specimen in the orthogonal test method, the average value of the ultimate 28-day strain index of each factor of the CNT incorporation rate is obtained, and the influence of CNTs on the ultimate strain of CNTFC is analyzed, as shown in Figure 11.

The ultimate strains of the CNTFCs under tensile and compressive pressures first increased and then decreased, and the trends were basically the same; however, the change after the ultimate strain exceeded 0.1 was not obvious. When the incorporation rate of CNTs increases from 0 to 0.05%, the ultimate compressive strain increases by 17.62%, and the ultimate tensile strain increases by 20.91%. When the incorporation rate of CNTs increases from 0.05% to 0.10%, the ultimate compressive strain is reduced by 28.7%, and the ultimate tensile strain is reduced by 28.50%. From 0.10% to 0.15%, the ultimate compressive strain continues to decrease by 1.966%, and the ultimate tensile strain decreases by 0.691%, which is a small decrease. CNT network integrity replaces conductivity data, based on the correlation of “CNT content-specific strength” (Figure 10). The 0.05% CNT group: the specific strength is 8.9 N·m/g, which is 1.44 times that of the group without CNTs (6.2 N·m/g), indicating that the CNT network is intact without agglomeration; 0.10% CNT group: the specific strength is 7.8 N·m/g, decreasing by 12.4%, corresponding to slight agglomeration; 0.15% CNT group: the specific strength is 7.2 N·m/g, decreasing by 19.1%, corresponding to severe agglomeration. This is consistent with the law that “conductivity decreases as the degree of CNT agglomeration increases” [17], and it can replace conductivity data to characterize CNT network integrity. CNTs improve the mechanical properties of a specimen by virtue of their excellent tensile properties at the nanoscale, effectively suppressing the initiation and expansion of cracks; therefore, CNTs have a significant effect on the improvement of the ultimate strain. When the incorporation of CNTs is 0.05%, the ultimate strain lifting effect is the most obvious. Continuing to increase the blending amount will reduce the ultimate strain. Excessive CNT agglomeration increases the dielectric transition region and reduces the overall tensile properties of CNTFCs. After the incorporation of CNTs exceeds 1.04%, a small decrease is still exhibited, indicating that the agglomeration transition zone has formed a weak region of CNTFC, which is reflected in the tensile strength of the fiber interlaced group. A continuous increase in the amount of CNTs incorporated does not change the mechanical properties of the fiber agglomeration zone, which is not necessary from a cost perspective.

#### 3.5.2. Microanalysis

The SEM test was carried out on the #6 specimen with the highest 2:1 cylinder strength and the #8 specimen with the highest split tensile strength in the orthogonal test, as shown in Figure 6. During curing, the concrete has many pores. Hydrated products and CNTs crisscross to form a network that runs through its pores, enhancing pore structure connectivity. Although the pores are abundant, the intertwined complex network structure connects the cement with the aggregate and effectively increases the strength of the CNTFC at a lower density level. Due to the introduction of a large number of bubbles, the pore structure between the particles further develops, and a distinct throughgoing crack is formed in the resultant stress field. As shown in Figure 6, in the #6 specimen and the #8 specimen, a large amount of acicular, layered and flake hydrates are observed at a 200 nm scale. CNT fibers are clearly seen interspersed between cement bodies, forming bridges. Intertwined CNT fibers interweave with hydrates into a spatial network structure, filling voids. CNT incorporation complicates the propagation path of CNTFC nanoscale cracks. This makes the material absorb more external energy during crack expansion, and it improves the macroscopic mechanical properties of CNTFC. It also effectively enhances its overall strength. As shown in Figure 6a, the proportion of CNTs in the #6 specimen is 0.15%. The probability of the fibers overlapping and agglomerating is greatly increased over that of other specimens. The dispersion in the matrix is further decreased, and the agglomeration and nucleation efficiency are apparent. The bridging effect is weakened, and larger fiber agglomerates occupy a continuous space, which expands the transition zone of the medium. Too many interfacial transition zones cannot fully exert the effect of the fiber inhibiting cracks, and the strength will be weakened. As shown in Figure 6b, the CNT content in eight specimens is 0.05%, the CNT fibers are well distributed and the nucleation and bridging effects are fully exerted. The unevenly distributed CNT fibers constitute interlayer dislocations and defects. In turn, the CNTFC has both a certain strength and damping performance, achieving good compression and splitting strength.

The SEM test is carried out on the #13 specimen with the lowest compressive and splitting tensile strength from the orthogonal test, as shown in Figure 7. At the 2 μm scale, the cracks and pores are more abundant, and the crystal integrity is poor. A large number of very small inert particles are distributed around the colloid without being wrapped, resulting in a low density and strength. In the case of a high FA incorporation rate, high bubble content and low cement content, 0.05% incorporation of CNT fibers is difficult to interlace into a network in the case of excessive foam pores and less gelled hydration products due to the overall structure; therefore, the specimen has a lower density, a lower strength, and a lower specific strength index.

## 4. Comprehensive Analysis and Determination of the Material Ratio of Buffer for Surrounding Rock Support

Table 4 shows that the 2:1 cylinder strength and splitting tensile strength of the #1, #6 and #8 specimens are the highest. The 2:1 cylinder strength ranges between 12.095 MPa and 18.381 MPa, and the corresponding splitting tensile strength ranges between 1.210 MPa and 1.997 MPa. Taking the surrounding rock support of the transportation lane of the Baiyangling coal mine in Shanxi Province, China, as an example, the maximum supporting force of the high-strength anchor is 150 kN, and the anchoring pallet area is 0.040 m^2^, which needs to be achieved when the CNTFC is set under the local stress concentration of the supporting structure. The strength is 3.75 MPa. Considering the influencing factors, such as the stress concentration of the supporting structure, the safety factor is 1.2, and the CNTFC strength should be greater than 4.50 MPa. Considering the mechanical strength, dead weight and economic cost of the material used for surrounding rock support pressure relief, the densities of the #1, #6 and #8 specimens are all greater than 1300 kg/m^3^, although the strengths are sufficient and within the range of lightweight concrete requirements. However, because of its considerable weight, the proportion of FA incorporation is too small, according to Table 6, the overall economics are unfavorable. The #3, #4, #9, #10, #15 and #16 specimens meet the mechanical strength requirements, but the densities are greater than 1000 kg/m^3^, the proportion of CNT incorporation is higher and the economics are unfavorable. The #2, #5 and #7 specimens have low incorporation rates of FA, and the economics are unfavorable. The #11 and #12 specimens have an FA mixing ratio of 80% and a CNT incorporation ratio of 0~0.05%. The economics are favorable, and the mechanical indexes also meet the requirements of surrounding rock support and pressure relief. The characteristics of the stress–strain curves suggest the applicability of the pressure-relief material.

**Figure 12:**
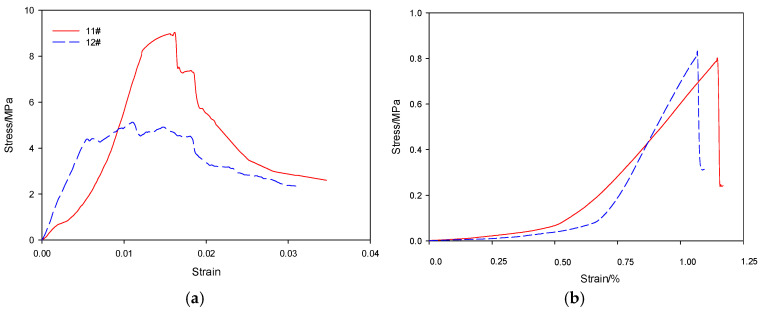
Stress–strain curves. (**a**) Uniaxial compression stress-strain curve; (**b**) Splitting tensile strength curve.

The stress–strain data of the #11 and #12 specimens are processed. The stress–strain data of each of the three specimens are controlled by the loading rate and the loading step. The control strain is constant, and the corresponding stress data are averaged and used to draw a stress–strain curve. The 2:1 cylinder compressive stress–strain curve is shown in Figure 12a, and the splitting tensile strength curve is shown in Figure 12b. Figure 12a shows that the peak compressive strength and ultimate strain of the #11 specimen are larger than those of the #12 specimen, and the elastic modulus of the #11 specimen is less than that of the #12 specimen, indicating that the #11 specimen at the early stage has deformation absorption capacity, and the #11 specimen can maintain a large deformation after the peak strength while still providing high resistance, which is a feature required to support the deformation of the surrounding rock. Figure 12b shows that the tensile strength of the #11 specimen is slightly lower, but it is not much different from the #12 specimen, and the ductility is obviously better than that of the #12 specimen. Although the #11 test specimen incorporates 0.05% CNTs, and the cost of the #12 test specimen is high, the mechanical properties are greatly improved while maintaining the same density. Therefore, it is determined that the #11 test specimen reflects the optimal mixing ratio of the roadway surrounding rock support for pressure relief. For the application scenario of the Shanxi Baiyangling Coal Mine roadway, its durability and behavioral risks must be comprehensively evaluated by considering the environments of high humidity, chemical corrosion, long-term static load and cyclic dynamic load. In terms of durability, high humidity can cause pore water penetration and damage the interfacial bonding between CNTs and hydration products. Acidic mine water and high CO_2_ can also cause sulfate expansion, chloride-induced interfacial degradation and carbonation embrittlement. In terms of behavioral risks, high porosity causes creep strain under long-term static load. Cyclic load cycles can cause repeated opening and closing of microcracks and fatigue fracture of CNT bridging structures. Cyclic load cycles cause repeated opening and closing of microcracks and fatigue fracture of CNT bridging structures, thus leading to a decrease in compressive strength and loss of ductility. The root cause of the above problems is the high foam volume fraction and high fly ash content. In engineering, it is necessary to optimize durability by adding waterproofing agents/silica fume, controlling the foam volume fraction, increasing the safety factor and verifying long-term reliability through field exposure tests. For specific engineering cases of surrounding rock support for pressure relief, specific force analysis and further testing between the #11 specimen and the #12 specimen mixing ratios should be carried out to determine the optimal ratio plan.

## 5. Conclusions

Underground mine roadways, tunnels and other soft rock engineering projects have requirements for support and pressure-relief materials: “lightweight, high mechanical properties, low cost”. Traditional foamed concrete, though lightweight, environmentally friendly and energy-absorbing, has insufficient mechanical properties, and it is difficult to meet soft rock support requirements. Carbon nanotubes (CNTs) have excellent performance, providing a new direction for improving their performance. This study aims to prepare CNT-modified foamed concrete (CNTFC) suitable for pressure relief in soft rock surrounding rock support. It is hypothesized that microdosage CNTs (0~0.15%, based on the total mass of cementitious materials) can form a nano-network with the hydration products of cement and fly ash while slightly increasing density and enhancing performance, and there exists an optimal dosage. To verify the hypothesis, this study adopts a five-factor, four-level orthogonal test (fly ash content, sand–cement ratio, water–binder ratio, CNT content, foam volume fraction). Combined with mechanical tests, stress–strain analysis and SEM microscopic characterization of specimens after 28 days of curing, the optimal mix ratio was screened out to meet the roadway support requirements of the Baiyangling Coal Mine in Shanxi Province (strength ≥ 4.5 MPa). In summary, the study draws the following core conclusions:

(1) The foam volume fraction, water–binder ratio and aggregate–cement ratio are the top three factors affecting the strength of CNTFC. The influence of the CNT incorporation rate and the FA incorporation rate on CNTFC strength are the next two most important factors.

(2) Among the five factors of FA incorporation rate, aggregate–cement ratio, water–binder ratio, CNT incorporation rate and foam volume fraction, though fly ash (reducing cementitious material cost by 33%) and foam (accounting for less than 3% of the total cost) can reduce concrete cost, they significantly deteriorate mechanical properties (when fly ash content increases to 100%, the 2:1 cylinder strength decreases by 27.40%; when the foam volume fraction increases to 150%, the strength decreases by 54.2%). Increasing the water–binder ratio also reduces strength. Only the optimal sand–cement ratio and CNT content can improve performance. Among them, the optimal CNT content is 0.05% (compressive strength increases by 15.3%, splitting tensile strength increases by 6.5% and density increases by only 2.0%). The optimal mix ratio adapted to the Baiyangling Coal Mine in Shanxi Province not only meets the engineering strength requirements but also reduces the cost by about 28% compared with the higher-strength mix ratio.

(3) CNTs improve the mechanical properties of a specimen by improving its tensile properties at the nanometer scale. At the optimal dosage of 0.05%, uniaxial compressive strength increases by 15.3%, and splitting tensile strength increases by 6.5%. Specific fracture energy increases from 86 J/m^2^ to 128 J/m^2^, specific strength reaches 8.9N·m/g, density increases by only 2.0% and ultimate compressive/tensile strains increase by 17.62% and 20.91%, respectively. This effectively inhibits the initiation and expansion of cracks, but excessive blending will decrease the strength and ductility of the foamed concrete.

(4) The optimum proportion of CNTs in CNTFC is 0.05%, with a proper FA, cement and foam blending ratio to fully realize the performance of the CNT fiber.

(5) The specific mixing ratio of a CNTFC used for pressure-relief support of surrounding rock should be considered with the specific engineering conditions and the costs. The paper takes the pressure-relief support of the surrounding rock of the Baiyangling coal mine in Shanxi Province, China, as an example, determining that the #11 CNTFC specimen is optimal: the corresponding FA incorporation rate is 80%, aggregate–cement ratio is 10%, water–binder ratio is 45%, CNT incorporation rate is 0.05% and foam volume fraction is 150%.

## Figures and Tables

**Table 1:** Material parameters of CNTs [23].

Material Type	Morphology	Purity (%)	Grey (%)	Diameter (nm)	Tube Length (μm)	Specific Surface Area cm^2^/g	Bulk Density (g/cm^3^)
MWCNTs	fluffy black powder	>97	<2.5	3–15	15–30	250–270	0.06–0.09

**Table 2:** Chemical compositions of materials (mass fraction %) [26].

Chemical Compositions	Fly Ash	Cement
SiO_2_	53.75	22.56
Al_2_O_3_	29.37	4.64
CaO	3.68	61.28
Fe_2_O_3_	5.64	2.35
MnO	1.08	2.04
Na_2_O	_	0.60
K_2_O	0.68	0.75
SO_3_	1.29	2.83
LOSS	3.58	2.01

**Table 3:** Horizontal table of orthogonal factors [35].

	Factors	A FA Incorporation Rate/%	B Aggregate–Cement Ratio/%	C Water–Binder Ratio/%	D CNT Incorporation Rate/%	E Foam Volume Fraction/%
Level						
1		40	0	45	0	50
2		60	5	50	0.05	80
3		80	10	55	0.10	120
4		100	15	60	0.15	150

Note: The foam content is the volume fraction, and the others are mass fractions.

**Table 4:** Orthogonal test table and mechanical test results.

	Category	A	B	C	D	E	2:1 Cylinder Strength /MPa	Splitting Tensile Strength/MPa	Density/ g/cm^3^
Number									
#1		1	1	1	1	1	12.853	1.921	1.370
#2		1	2	2	2	2	5.427	0.892	0.920
#3		1	3	3	3	3	6.579	0.574	1.010
#4		1	4	4	4	4	7.862	0.705	1.030
#5		2	1	2	3	4	3.880	0.441	0.760
#6		2	2	1	4	3	18.381	1.210	1.350
#7		2	3	4	1	2	4.344	0.405	0.710
#8		2	4	3	2	1	12.095	1.997	1.380
#9		3	1	3	4	2	3.311	0.593	1.020
#10		3	2	4	3	1	14.054	1.871	1.010
#11		3	3	1	2	4	8.835	0.786	0.980
#12		3	4	2	1	3	5.127	0.804	0.930
#13		4	1	4	2	3	2.382	0.390	0.690
#14		4	2	3	1	4	2.605	0.724	0.900
#15		4	3	2	4	1	11.663	0.909	1.250
#16		4	4	1	3	2	6.094	1.122	1.020

**Table 5:** Orthogonal test range.

Types	*A* Fly Ash Incorporation Rate (%)	*B* Bone Glue Ratio (%)	*C* Water–Cement ratio (%)	*D* CNT Doping Rate (%)	*E* Foam Volume Fraction (%)
2:1 Cylinder strength	3.989	4.507	5.394	4.074	7.872
Splitting tensile strength	0.235	0.513	0.501	0.165	1.010
Density	1.175	1.225	3.275	2.200	3.275

**Table 6:** Price list.

Raw Materials	Specification Attributes	Market Unit Price (Euro/Kilogram, €/kg)	Cost Affects Key Features
Cement	PO42.5ordinary Portland cement	0.15	Gelling core, with a unit price higher than that of fly ash, its proportion directly affects the cost.
Fly ash (FA)	Grade I, fineness 10.8%	0.08	Industrial waste, as a substitute for cement, can reduce gelling costs by 33%
Multi-walled carbon nanotubes (CNTs)	Purity > 97%, surface grafted –COOH/–OH	180.00	The unit price is 1200 times that of cement, and the dosage is extremely sensitive to cost.
Natural sand	Particle size < 2.36 mm, apparent density 2.23 g/cm^3^	0.06	The proportion of aggregate cost is low (<5%)
ZT-F50 foam	Active substance 30%, initiation amount 60 mL/g	2.50	Added by volume (50%~150%), with a small total dosage

Cost per cubic meter of CNTFC (€/m^3^) = Amount of each raw material × The sum of the corresponding unit prices. Raw material consumption (kg/m^3^) = CNTFC measured density (kg/m^3^) × Mass fractions of raw materials (such as FA incorporation rate, CNT incorporation rate). Foam is a volume fraction, calculated as “foam volume / mixture volume = 1:1~1:1.5” conversion, with a small amount used and the cost accounting for less than 3%.

## Data Availability

The original contributions presented in this study are included in the article. Further inquiries can be directed to the corresponding author.
